# Perceived barriers to assessing understanding and appreciation of informed consent in clinical trials: A mixed-method study

**DOI:** 10.1017/cts.2021.807

**Published:** 2021-06-26

**Authors:** Erin D. Solomon, Jessica Mozersky, Kari Baldwin, Matthew P. Wroblewski, Meredith V. Parsons, Melody Goodman, James M. DuBois

**Affiliations:** 1 Bioethics Research Center, Washington University School of Medicine, St. Louis, MO, USA; 2 School of Global Public Health, New York University, New York, NY, USA

**Keywords:** Informed consent, assessment, validated assessments, research ethics, implementation science

## Abstract

**Introduction:**

Participants and research professionals often overestimate how well participants understand and appreciate consent information for clinical trials, and experts often vary in their determinations of participant’s capacity to consent to research. Past research has developed and validated instruments designed to assess participant understanding and appreciation, but the frequency with which they are utilized is unknown.

**Methods:**

We administered a survey to clinical researchers working with older adults or those at risk of cognitive impairment (*N* = 1284), supplemented by qualitative interviews (*N* = 60).

**Results:**

We found that using a validated assessment of consent is relatively uncommon, being used by only 44% of researchers who had an opportunity. Factors that predicted adoption of validated assessments included not seeing the study sponsor as a barrier, positive attitudes toward assessments, and being confident that they had the resources needed to implement an assessment. The perceived barriers to adopting validated assessments of consent included lack of awareness, lack of knowledge, being unsure of how to administer such an assessment, and the burden associated with implementing this practice.

**Conclusions:**

Increasing the use of validated assessments of consent will require educating researchers on the practice and emphasizing very practical assessments, and may require Institutional Review Boards (IRBs) or study sponsors to champion the use of assessments.

## Introduction

The ability to make informed decisions about research participation is fundamental to the ethical premise of respect for autonomy [1]. In line with this premise, federal regulations require that participants have adequate information before deciding on research participation and that a participant’s informed consent be obtained prior to participating [1, 2]. Making a decision to participate in research generally involves four parts: understanding, appreciation, reasoning, and expressing a clear choice [1, 3]. Understanding is the ability to understand or know the meaning of information presented, while appreciation is the ability to recognize how information is relevant to oneself [1, 3]. Reasoning is using the information to weigh options, and expressing a choice is the ability to clearly express a decision [1, 3]. All four components are essential to the consent process and ensure that participants can use consent information to make a decision in line with their own preferences [3, 4].

A challenge to informed consent in research settings is that participants and research professionals often overestimate how well participants understand consent information and experts often vary in their determinations of a participant’s capacity to consent [5–8]. There are numerous reasons why participants fail to understand and appreciate consent information. They may have a cognitive impairment, caused by neurological, psychiatric, or other medical diagnoses [9–15]. Older adults (age 65+), regardless of diagnoses, are at increased risk of cognitive impairment [16, 17]. Furthermore, participants with cognitive impairment may find their ability to understand consent information changes from one day to the next. It is also possible that the consent information was presented in an unclear manner, perhaps using dense prose and technical or legal language. Language proficiency may pose another barrier [18]. Finally, research participation is often offered following a new diagnosis, when the participant may be already feeling overwhelmed with information [19].

The National Institutes of Health recently put forth the Inclusion Across the Lifespan policy, which mandates that older adults be included in research unless there is a scientific or ethical reason to exclude them [20]. Older adults have routinely been excluded from clinical research, often because they are at higher risk of cognitive impairments, and therefore may face challenges with informed consent [16, 17]. As more older adults are necessarily included in research in the coming years, the odds of enrolling participants with cognitive impairments increases. Having procedures in place to determine participants’ level of consent understanding and appreciation will therefore become essential.

One method for ensuring participant understanding and appreciation is to use a validated informed consent assessment instrument. Some assessments are specific to one domain (e.g. cancer or drug trials) [21, 22] or present a hypothetical study to which the participant must provide the correct answers about the study [23]. Others assess participants’ understanding and appreciation of the study they are being asked to participate in [8, 24]. Assessments may take anywhere from 5 min to 20 min [8, 23].

Assessing participants’ understanding and appreciation in clinical trials has several benefits. First, it ensures that all participants understand and appreciate the information presented to them during the consent process and removes the need for researchers to rely only on their clinical judgment. Second, it helps to identify participants who may require additional education or clarification regarding aspects of the research. Research is often complex and unfamiliar to participants, and they may need to have parts of the study explained to them more than once in order to fully understand and appreciate it [25, 26]. Third, it can identify weaknesses in the consent process. For example, if participants tend to misunderstand the same piece of information, perhaps the consent process needs modification. Fourth, assessing all participants avoids unfairly targeting or stigmatizing those with certain diagnoses or other characteristics (such as dementia, schizophrenia, etc.) by presuming their ability to understand the information is limited. Fifth, it may expedite IRB approval by reducing concerns about the informed consent process (the most common concern voiced by IRB members) [27]. Last, it can identify participants who may need a Legally Authorized Representative (LAR) to consent on their behalf. Participants who continue to demonstrate inadequate understanding, even after additional education is provided, may require assistance with decision making. In such cases, the participant may need to provide their assent, and lower scores on an assessment may be acceptable for this purpose.

There is no regulatory standard for when to use an assessment of consent understanding and appreciation, and IRB guidance can vary [28]. Furthermore, there are no published data on how frequently researchers assess consent understanding and appreciation, or when it would be appropriate to do so. For example, trials involving only minimal risk—such as a single blood draw—may not require an assessment due to their relative simplicity. However, as the level of risk of a study increases, so does the need to determine and document that participants have understood the information before deciding to take part [28].

The current research was part of a larger implementation science project focused on increasing the use of evidence-based informed consent practices among researchers in the USA (NIA R01AG058254). The data from this study will inform a trial that ultimately seeks to increase adoption (i.e., implementation) of these practices, one of which is using validated consent assessments. We utilized the Consolidated Framework for Implementation Research (CFIR) to guide the overall project [29, 30]. CFIR is composed of five domains: a) the characteristics of individuals who are targeted to implement the new practice, [31, 32] b) their outer setting (e.g., Office of Human Research Protection regulations or study sponsors) [32–35], c) their inner setting (e.g., their local IRB or study protocols) [32, 34, 36], d) the characteristics of the intervention being implemented [32], and e) the process of implementation [32, 37]. To our knowledge, this will be the first implementation science trial conducted within the domain of research ethics and informed consent.

For the current research, we sought to better understand three broad questions pertaining to the use of validated consent assessments.

1. How widespread is the use of validated consent assessments? We seek to understand the current rates of adoption to establish a baseline rate of use.
2. What modifiable factors predict adoption of validated consent assessments? We seek to understand the modifiable factors associated with adoption of validated assessments. This will yield important information about what interventions might increase researchers’ adoption of the practices for our upcoming implementation trial. We defined modifiable as any factor that could be targeted for change in our upcoming trial. For example, attitudes are potentially modifiable, but funding sources are not.
3. What are the perceived barriers to adopting validated consent assessments? Understanding the perceived barriers to adoption will help determine how to improve researchers’ use of validated assessments.

## Materials and Methods

We used a mixed-methods approach, collecting both quantitative survey data and qualitative interview data, to gather a more complete picture of how often validated assessments are used and the perceived barriers to using them [38]. This research was approved by the Washington University in St. Louis IRB (#201807033 and #201909154). The study samples consisted of principal investigators (PIs), clinical research coordinators (CRCs), and (in the qualitative interviews only) IRB members. PIs were included in the samples because even though they often do not obtain consent, they are ultimately responsible for how their trials are conducted and have the ability and authority to make changes to the consent process. CRCs were included in the samples because they obtain consent. IRB members were included in the qualitative interview sample because they are an integral aspect of the ethical conduct of research, and all consent procedures have to be approved by the IRB. Thus, their views were a particularly valuable addition to the research.

### Quantitative Survey

#### Survey development

PhD-level experts in the fields of research ethics, bioethics, and survey design wrote all items with expert input from PIs, CRCs, and IRB members [39–42]. We modified some items to create a PI version and CRC version where relevant. The survey focused on multiple evidence-based consent practices, and we present here only the data on using validated assessments of consent. Cognitive interviews on the survey items were conducted with individuals with expertise in informed consent regulations, conducting consent procedures, and/or designing consent protocols (*N* = 8). Following the cognitive interviews, items were revised to improve clarity and reduce overall survey length and burden.

#### Measures

The survey instrument explored the use of assessments, perceived barriers to using assessments, attitudes toward assessments, and confidence in having the resources needed to use assessments. We also measured social desirability as a control variable and collected demographic information.

##### Personal adoption

To measure personal adoption of the practice, we first presented a short description of validated consent assessments to ensure that all participants were aware of what they were:

*[One consent] practice is assessing participants’ understanding and appreciation of informed consent information using a validated assessment tool. Such tools often involve scoring participant responses to questions about the consent form to determine whether they understand the study and what they are being asked to do. Validated assessments are ones that have been peer reviewed and published*.

We then asked how many clinical trial protocols they had submitted to an IRB that included an intervention of greater than minimal risk in the past year, and how many of those protocols they *personally modified* to add a validated assessment. Adoption was calculated by dividing the number of protocols they had added a validated assessment to by the number of greater-than-minimal-risk intervention protocols they had submitted to the IRB in the past year. After multiplying by 100, personal adoption is expressed as a percentage.

##### Reasons for not adopting

Participants who did not report using the practice at all were presented with a list of options as to why they did not use the practice (e.g., “*I did not think this practice was important,” “I was unaware of this practice”*).

##### Change already made

Two of the reasons for nonadoption, “*My research team, group, or lab already uses this practice”* and, “*The sponsor already required it*,” indicated that a participant did not have an opportunity to adopt the practice because adoption had already occurred. If either of these response options were endorsed, the participant was considered to have already adopted the practice, although they had not personally made the change. When combined with the personal adoption rate, this yielded an overall adoption rate.

##### Barriers

All participants, regardless of whether they had reported using the practice, were asked if anyone might prevent them from implementing it. If they responded *“yes,”* they were presented with a list of options (*i.e., “IRB,” “sponsor,” “participants,” “research team members,”* and *“other”*).

##### Positive attitudes

We measured attitudes toward using validated assessments of consent with two questions, “*How useful do you think this practice is in enhancing research participants’ understanding of consent information?*” (1 = *not at all useful*, 5 = *extremely useful*) and “*How interested are you in improving your use of this practice?*” (1 = *not at all interested*, 5 = *extremely interested*). We summed the responses to these items to produce a positive attitudes score that ranged from 2 to 10.

##### Confidence in resources

We asked participants “*How confident are you that you have the resources you need to use this practice well?*” (1 = *not at all confident*, 5 = *extremely confident*).

##### Marlowe–Crowne Social Desirability Scale

The short form version of the Marlowe–Crowne Social Desirability Scale was used to measure social desirability [43]. The scale generates a score range of 1 – 13 with higher scores indicating a higher desire for social approval [43]. The scale has a KR20 reliability score of .88; [43] in the current study, the KR20 was .67.

##### Demographics

We collected data on participant’s gender, age, race, education, and information about trials they worked on.

#### Survey participants

We recruited the quantitative survey participants (*N* = 1284) using nonprobability, criterion-based sampling. We targeted researchers whose participants have cognitive impairments or whose studies are open to older adults (age 65+) because they are at higher risk for cognitive impairment [16, 17]. We targeted researchers working in the USA because regulations on informed consent vary importantly across nations.

We used two methods to recruit survey participants. First, we created a recruitment database by querying the Aggregate Analysis of ClinicalTrials.gov (AACT) database which houses publicly available information about clinical studies [44]. The database included 20,613 researchers working on interventional clinical trials focused on Alzheimer’s disease (527) or involving participants age 65 or older (20,086). All participants were then contacted via E-mail. Second, we posted a recruitment message to the Association of Clinical Research Professionals (ACRP) social media groups (i.e., Facebook and LinkedIn) and sent two recruitment E-mails to 9,774 of ACRP’s E-mail list members. In each of these recruitment methods, a link to our online Qualtrics survey was provided. Participants provided their informed consent prior to completing the survey and received a $20 Amazon eGift Card for participating.

We screened participants to verify that they were a CRC or PI, worked in the United States, and expected to be involved in at least one new clinical intervention trial that would open within the next 18 months. CRCs were also asked if they prepared informed consent documents, assisted in preparing consent documents, or obtained informed consent from participants in clinical trials that involve interventions. We removed from the data any participants who screened out (*N* = 618) or completed the survey multiple times (*N* = 27). Participants who completed the survey in under five minutes or provided impossible responses to more than one of the consent practice adoption items (i.e., claiming to have added a consent practice to more protocols in the past year than they had submitted to an IRB in the past year) were also removed from the study (*N* = 67) [45]. Thirty-one cases were retained in the data set as a whole, but excluded from analyses using personal adoption of assessments for providing an impossible response to that item.

### Qualitative Interviews

#### Interview guide development

Interview questions were developed using the CFIR model as a framework. Each stakeholder group interviewed (i.e., PIs, CRCs, and IRB members) had their own semi-structured interview guide with adapted open-ended questions; however, each interview followed a similar format. Participants were asked about their current informed consent practices and their attitudes toward evidence-based consent practices. Validated consent assessments were described in the interview guide using similar language used in the quantitative survey. The interviews were conducted prior to administering the quantitative survey.

#### Interview participants

We interviewed PIs, CRCs, and IRB members (total *N* = 60). PIs (*N* = 20), and CRCs (*N* = 20) were identified through trial listings on ClinicalTrials.gov. We used purposive sampling to ensure that the sample represented researchers conducting trials with Alzheimer’s disease patients (CRCs 80%, PIs 75%). IRB members (*N* = 20) were identified through the websites of the 32 US institutions with an NIA Alzheimer’s Disease Research Center (ADRC) [46] and through institutions that were a part of the American Association of Medical Colleges (AAMC) [47]. IRB members needed to be voting members of their IRB and to have reviewed at least one clinical trial protocol involving older adults or individuals with cognitive impairments in the past year in order to participate.

Participants were recruited via E-mail, provided informed consent, and completed a demographic survey. Participants then completed a one-hour, semi-structured telephone interview and received a $40 Amazon eGift Card for participating. All interviews were audio recorded and professionally transcribed.

### Data Analysis

We used SPSS version 26 and Stata 16 to analyze the quantitative survey data. For Research Question 1 (rates of adoption), we calculated the mean percentage of personal adoption and the total number of participants who had used the practice. We used regression analyses for Research Question 2 (predictors of adoption). We entered the Marlowe–Crowne Social Desirability scale and the “change already made” variable into block 1 of the regression to account for socially desirable responding and for those who did not have the opportunity to personally modify their protocol and adopt the practice. We identified variables that could be modified by an intervention and entered them into block 2 of the regression. These variables were barriers, positive attitudes, and confidence in resources. To keep the regression models parsimonious and to keep with our focus on informing implementation efforts, we did not include variables that are unable to be influenced, such as funding source. Analysis for Research Question 3 (barriers to adoption) involved tallying the percentage of survey participants indicating various reasons for not adopting the practice and types of barriers reported.

We used Dedoose software to analyze the qualitative interview transcripts. We used a mixture of inductive and deductive coding, using CFIR to guide our codebook development [48]. Each stakeholder group was assigned one gold standard coder, and coders (KB and ES) were trained on the codebook. Coders were required to attain a Cohen’s kappa score at or above .80 before coding the data. Cohen’s kappa was calculated a second time mid-way through coding to prevent drift. During the coding period, the coders met weekly to resolve questions, and the team revised the codebook accordingly.

## Results

Table 1 presents demographic results for the survey (*N* = 1284) and interview samples (*N* = 60).

**Table 1.** Demographic characteristics of quantitative survey and qualitative interview samples

		Qualitative Interviews		
	Quantitative Survey	PI	CRC	IRB
Variable	%	%	%	%
Gender				
Female	77	65	85	55
Male	22	35	15	45
Other	<1	0	0	0
Prefer not to answer	1	0	0	0
Age				
Below 30	17	0	35	0
30–39	33	25	45	15
40–49	26	30	10	25
50 or more	24	45	10	60
Race/ethnicity^a^				
American Indian/Alaska Native	1	0	0	0
Asian	9	20	5	5
Black/African American	5	0	5	0
Hispanic or Latino	9	5	5	0
Native Hawaiian/Pacific Islander	<1	0	0	0
White	83	75	90	90
More than one race	3	0	0	0
Prefer not to answer	2	15	5	5
Education				
High School Diploma or GED	3	0	0	0
Associate’s Degree	6	0	0	0
Bachelor’s Degree	38	0	35	0
Master’s Degree	31	0	50	15
Doctoral Degree	20	95	10	80
Other	2	5	0	5
Trial types^a^				
Drug	76	45	65	85
Device	48	5	20	90
Behavioral	31	60	70	90
Biologics	25	10	20	65
Surgical	24	0	15	75
Funding sources^a^				
Federal agencies	65	80	80	100
Private foundations	36	50	35	85
Industry	75	65	50	85
Other	9	5	10	20
Submitted ≥1 greater-than-minimal-risk protocol in prior year	73			
≥1 clinical trial open to older adults	99			
≥1 clinical trial involving participants with cognitive impairments	34			
Implemented an assessment at least once in past year ^b^	44			

*Note.* Quantitative survey sample *N* = 1284 (232 PIs and 1052 CRCs). Qualitative interview sample *N* = 60 (20 PIs, 20 CRCs, and 20 IRB members). ^a^Participants could select more than one response. ^b^ *N* = 936 for this variable only, because only 936 participants had an opportunity to adopt the practice by submitting at least 1 greater-than-minimal-risk protocol to the IRB in the past year and had a valid personal adoption score. PI, principal investigator; CRC, clinical research coordinator; IRB, institutional review board.

### Research Question 1: Current Adoption Rates

**Fig. 1.**
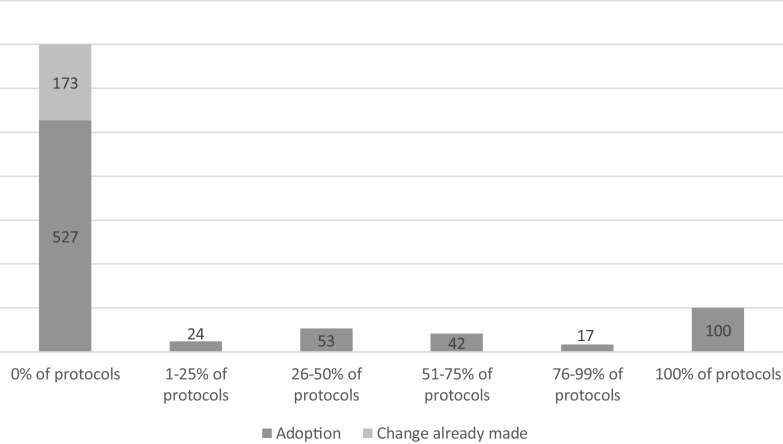
Frequency of Assessment Adoption among Quantitative Survey Sample Participants Who Submitted ≥ 1 Greater-Than-Minimal-Risk Protocol in the Prior Year (N = 936). *Note.* Numbers in figure are number of participants falling into each range of the adoption variable, and how many of the non-adopters reported that the change had already been made by either the study sponsor or another member of their research team. Only 73% (*N* = 936) of the sample is represented here because only 73% had submitted at least 1 greater-than-minimal-risk protocol to the IRB in the past year (which was the denominator for the adoption variable calculation) and had a valid personal adoption score (i.e., 0–100%).

Table 2 presents means and standard deviations for continuous variables of interest. Most participants (73%; *n* = 936) had submitted at least one protocol of greater than minimal risk to the IRB in the past year, providing an opportunity to modify a protocol to adopt an assessment. Of those with an opportunity to adopt, 25% (*n* = 236) of participants had personally modified at least one protocol to include a validated assessment in the past year, while the majority, 75%, (*n* = 700) did not (see Fig. 1). Of those nonadopters (*n* = 700), 25% (*n* = 173) reported that either someone else on their research team, group, or lab had already made the change or their sponsor already required it. After combining the personal adopters (*n* = 236) with the participants who reported that the change had already been made (*n* = 173), this meant that 44% of participants had implemented an assessment at least once in the past year. This means that 56% (*n* = 527) of participants with an opportunity to adopt did not use validated assessments at all in the past year. Among those who did modify protocols to include an assessment, they did so an average of 72% of the time. In other words, they personally modified 72% of the greater-than-minimal risk protocols they submitted to the IRB in the past year.

**Table 2.** Means and standard deviations of quantitative survey sample

Variable	*M*	*SD*	Range
Number of greater-than-minimal-risk trials submitted to IRB submitted in past year	4.46	7.31	0–90
Number of trials modified to add an assessment	1.15	3.14	0–34
Confidence in resources	3.11	1.21	1–5
Positive attitudes	6.97	1.92	2–10
Marlowe–Crowne Social Desirability	9.19	2.47	1–13

*Note. N* = 1284. *Mean percentage for adoption rate is the average percent of trials modified to add an assessment, out of the total number of trials of greater than minimal risk they submitted to an institutional review board (IRB) in the past year. *N* = 936 for adoption rate because only 936 submitted ≥ 1 greater-than-minimal-risk protocol to the IRB in the past year indicating an opportunity to adopt and had a valid personal adoption score (i.e., 0–100%)

### Research Question 2: Predictors of Adoption

As seen in Table 3, the overall regression model in block 2 was significant, adj. *R* ^*2*^ = .18, *p* < .001. Results from block 2 show that not seeing the sponsor as a barrier, having more positive attitudes, and having more confidence in resources were all significant predictors of adopting validated assessments. This is a mixture of CFIR outer domain (sponsor) and individual domain (attitudes and confidence) variables.

**Table 3.** Regression analyses predicting adoption in the quantitative survey sample

Block	Variable	*B*	*β*	*t*	*p*	*F*	*df*	*adj. R* ^*2*^
1	Overall model				<.01	32.52	2, 933	.06
	Marlowe–Crowne	−.01	−05	−1.44	.15			
	**Change already made**	−**.22**	−**.25**	−**7.72**	**<.01**			
2	Overall model				<.01	26.57	8, 927	.18
	**Marlowe–Crowne**	−**.01**	−**.10**	−**3.20**	**<.01**			
	**Change already made**	−**.29**	−**.32**	−**10.43**	**<.01**			
	IRB as barrier	−.03	−.02	−.68	.50			
	**Sponsor as barrier**	−**.08**	−**.07**	−**2.04**	**.04**			
	Team members as barrier	<.01	<.01	.02	.98			
	Participants as barrier	.05	.02	.78	.44			
	**Positive attitudes**	**.01**	**.07**	**2.27**	**.02**			
	**Confidence in resources**	**.09**	**.31**	**9.09**	**<.01**			

*Note. N* = 936. The dependent variable was adoption of validated assessments of consent in the past year. Bolded variables were significant predictors of adoption. IRB, institutional review board.

### Research Question 3: Reasons for Not Adopting and Barriers to Adoption

#### Quantitative survey

As seen in Table 4, the most common reasons for not adopting a validated assessment were “*I was unaware of this practice,” “I’m not sure how to do this,”* “*The sponsor already required it,”* and *“My research team, group, or lab already uses this practice.”* One hundred and seventeen participants indicated “*other.”* Responses to our open-ended follow-up question indicate that they gauge their participants’ understanding informally without a validated tool, they were not responsible or not allowed to make this type of change to their protocols, or their sponsor or IRB may not allow this practice.

**Table 4.** Reasons assessments were not adopted and perceived barriers to adoption in quantitative survey sample

Survey question	N	% of question respondents	% of sample
Reasons for non-adoption	700		
I was unaware of this practice (CFIR individual)	320	45.7	24.9
I’m not sure how to do this (CFIR individual)	130	18.6	10.1
The sponsor already required it*	98	14.0	7.6
My research team, group, or lab already uses this practice*	93	13.3	7.2
I did not think using a validated assessment was important (CFIR individual)	48	6.9	3.7
I do not have time to make optional changes to study protocols (CFIR individual)	25	3.5	1.9
I do not believe the IRB would allow this (CFIR inner)	24	3.4	1.9
I did not want to risk a delay in IRB review time (CFIR inner)	19	2.7	1.5
Other	117	16.7	9.1
Barriers to Adoption	258		
Research team members (CFIR inner)	134	51.9	10.4
Sponsor (CFIR outer)	114	44.2	8.9
IRB (CFIR inner)	104	40.3	8.1
Participants (CFIR inner)	52	20.2	4.0
Other	26	10.1	2.0

*Note.* For reasons for non-adoption, only participants reporting not adopting the practice answered the question (*N* = 700). For barriers to adoption, only participants reporting that someone might try to prevent them from using the practice answered the question (*N* = 258). Percentage of question responders is the percentage of participants who selected that response option out of those that answered the question. Percentage of sample is the percentage who selected that response option of the total number of participants in the quantitative survey sample (*N* = 1284). *Response option that comprises the change already made variable and does not represent a CFIR domain. Participants could select all response options that applied, and all response options are presented here. CFIR, Consolidated Framework for Implementation Research ; IRB, institutional review board.

In response to the item, *“Do you think anyone might try to prevent you from using this practice?”* 258 said “yes.” When asked who would prevent them, participants indicated “*research team members*” (52%), “*sponsor*” (44%), “*IRB*” (40%), and “*participants*” (20%). This is a mixture of CFIR inner domain (IRB, research team members, participants) and outer domain (sponsors) factors.

#### Qualitative interviews

Participants identified barriers and facilitators of adoption in the three relevant CFIR domains: individual characteristics, inner setting, and outer setting.

##### Individual characteristics

As seen in Table 5, the majority of PIs, CRCs, and IRB members reported positive opinions about using validated consent assessments (17 PIs, 18 CRCs, and 13 IRB); however, only 1 PI and 4 CRCs reported actually using one. Participants also expressed numerous concerns about validated consent assessments (17 PIs, 13 CRCs, and 7 IRB). Many of these concerns centered around the perception that participants may have negative feelings or reactions to being assessed (10 PIs, 8 CRCs, and 2 IRB) and doubting the value or trustworthiness of validated assessments (10 PIs, 2 CRCs, and 0 IRB). Other concerns included that using the practice might hurt enrollment, that it would not be needed for their study (often because they were already using an assessment of decisional capacity), or that they would need more training before they would be able to implement the practice. IRB members expressed the fewest concerns.

**Table 5:** Perceived barriers to adoption of practices indicated by qualitative interview participants

CFIR Domain	N of PIs	N of CRCs	N of IRB members	Representative Quotes
**CFIR Individual Characteristics**	20	20	18	
Statements of support	17	18	13	*I think the benefit is in the opportunity for the researcher to correct any misunderstanding, and…in seeing people’s understanding. Then if there’s an error and there’s a protocol such that the researcher has to go back and correct an error, reteach something, I think that overall, the understanding will improve.(PI11, Female, Age 60 or older, White, 27 years as PI)* *I hope that it’s a fairly common practice that people are asking and making sure that people understand, but this would take it one step further to ensure that that happened. (PI10, Female, Age 30–39, Asian, 2 years as PI)* *I would hope that every consent would kind of do something like this. Even for the ones that are straightforward just observational, why not have something that can assess your understanding as long as it doesn’t take up more time, or make it more burdensome, or things like that. (IRB13, Male, Age 40–49, Asian, 8 years as IRB)*
Statements of concern	17	13	7	
• Perceived participant dislike	10	8	2	*I think it would be too much pressure for patients to have to take a quiz right there, and I don’t know that they would be willing to participate. It might turn them off. (PI04, Female, Age 40–49, Asian, 14 years as PI)* *Well, the one thing is, is that I just think that you would put the study staff in a difficult situation doing it. I think that they could be trained, but again, I think it just raises this element of feeling like you’ve hurt a participant who wants to come into a trial, and you’re telling them, “I’m sorry. You don’t measure up.” (PI16, Male, Age 60 or older, White, 41 years as PI)* *It could also make patients a little bit more intimidated with the whole process (PI18, Male, Age 30–39, Asian, 2 years as PI).* *Some of the participants will feel really frustrated and get kind of angry or hostile. Sometimes, they’re just more shut down, depending on their personality, because, really, you’re playing out their deficits and you’re really pointing out their disease to them. It can be really challenging for them and really frustrating, and I would say that this could probably provoke that for them, too, and may make them irritable or sad, or just evoke some difficult emotions for them. (CRC01, Female, Age 20–29, White, 1 year as CRC)*
• Uncertainty and doubt about assessment instruments	10	2	0	*I don’t know if it would really help them understand the consent process better. I think it helps the team obviously understand if the person has the capacity to consent, but I don’t know if it would really help the participant per se. (PI01, Female, Age 40–49, White, 2 years as PI)* *I think you’d get wrong answers on the assessment that don’t reflect misunderstanding, but just reflect people not being able to take a test or put things in their own words. I don’t know what the assessment looks like, so I’m not sure it’s gonna necessarily measure understanding. (PI11, Female, Age 60 or older, White, 27 years as PI)*
Lack of Knowledge	8	8	11	*Is there actually a validated tool? Is this something that’s happening or is this something that may be under development? (PI10, Female, Age 30–39, Asian, 2 years as PI)* *I think it would take a lot of education and training for researchers and for IRB members. There would probably need to be some sort of template about the types of things that you had to have people tell you back what they understood. (IRB18, Female, Age 50–59, White, 2 years as IRB)* *I don’t know enough about this to know is there a standardized tool that you could use in all studies. I don’t know whether or not that exists. (IRB14, Female, Age 40–49, White, 11 years as IRB)*
**CFIR Inner Barriers**	18	13	18	
Burden	15	11	10	*Well, I think the time and the resources and determining what to be asked, and at what point should it be asked, and whether or not it’s a passing score. (PI03, Female, Age 40–49, race not disclosed, 9 years as PI)* *Our patients have long research days, and so I think for every piece of paperwork that you add in, it’s just gonna lengthen the overall consenting process. (CRC19, Female, Age 20–29, Asian, 2 years as CRC)* *I don’t know if participants would be confused with something like that just looking at the consent form questions we already have. I know sometimes the consent process itself seems a little overwhelming to participants, so I don’t know if that would add to it or not. (CRC08, Male, Age 20–29, White, 2 years as CRC)* *I think that any time you require implementation of any more advanced process it’s time consuming, and time is money, and that means people. If they have a research coordinator, it’s more time for the research coordinator, less time for something else. If they don’t have a research coordinator, it’s the investigator his or herself that has to do it. I think anytime you require something more advanced and time consuming it’s time. That’s a complaint I hear from everybody, “I just don’t have time to do this.” (IRB19, Male, Age 60 or older, race not disclosed, 16 years as IRB)*
Research team	3	1	10	*One [barrier] is that we have research teams who may say, “You know, actually, we have this process that we’ve been using for 10 years, and we find it is better than this validated tool.” (IRB06, Female, Age 30–39, White, 11 years as IRB)* *I think this practice would be most useful for study teams who don’t have an expertise in dementia, or who aren’t already familiar with and using other kinds of tools. If a neurology study team is already doing a comprehensive cognitive evaluation of a participant, they might resent being asked to add some additional assessment, rather than relying on the results of their own testing and expertise. (IRB04, Female, Age 40–49, White, 10 years as IRB)*

*Note*. Total *N* = 60 (20 PIs, 20 CRCs, and 20 IRB members). CFIR outer setting barriers were not included in the table because they were rarely mentioned by qualitative interview participants. CFIR, Consolidated Framework for Implementation Research; CRC, clinical research coordinator; IRB, institutional review board; PI, principal investigator.

We also identified a lack of knowledge as a barrier to adopting the practice (8 PIs, 8 CRCs, and 11 IRB). Individuals described their general unawareness of the practice, their relatively little experience with participants with cognitive impairments (assuming validated consent assessments were only for participants with cognitive impairments), needing training on how to administer the assessment, and what to do when participants provide incorrect responses, as barriers.

##### Outer setting barriers

The outer setting was rarely identified as a barrier, and only arose in 2 PI, 2 CRC, and 1 IRB member interview. Where mentioned, outer setting barriers included problems with accessibility for those whose first language is not English, and the difficulty of working with LARs in the event a participant fails a consent assessment.

##### Inner setting barriers

The most frequently cited inner barrier was burden (15 PIs, 11 CRCs, and 10 IRB). Burden primarily consisted of concerns about the time it would take to add an assessment to the already lengthy consent process, and the burden that extra time would place on participants. Some also indicated the time it would take to select an assessment, and the time it would take to train their team members on how to administer and score it. Notably, IRB members also tended to report the research team as a barrier (*N* = 10), which was not the case for PIs (*N* = 3) or CRCs (*N* = 1). IRB members reported that researchers would “push back” if they were asked to add an assessment to their consent procedures; some because they have been using the same procedures for a long time and are hesitant to change, and others because adding anything to an already long and complex process is undesirable.

## Discussion

This study was premised on the idea that using a validated assessment is appropriate in clinical trials that involve greater-than-minimal risk interventions when enrolling older adults or individuals with cognitive impairments. The reasons for this are manifold: routinely using validated assessments can improve the consent process (e.g., by identifying poorly explained material), identify individuals who may require special consent procedures (e.g., further education or surrogate decision-makers), and reduce stigma by making it routine. Additionally, doing this requires little training and time (e.g., 5 min for the UBACC [8]) and may offer additional benefits to researchers such as expediting IRB approval by reducing concerns about the informed consent process [27]. We found, however, that even in this special subgroup of trials, only a minority of clinical researchers (44%) used validated assessments in the past year and those who did use them did not use them consistently.

### Education Is Needed to Dispel Misconceptions

The most common reasons for not implementing assessments was a lack of awareness of the practice and being unsure how to administer an assessment. This suggests that education and training are needed to raise awareness and educate researchers on how to select and administer assessments. In qualitative interviews, PIs were also concerned about the assessment instruments themselves, doubting the validity of the instruments and whether they actually assess understanding. This suggests that PIs in particular will need to be educated on validity evidence supporting the use of assessments.

### IRBs and Sponsors May Need to Champion the Use of Assessments

Not seeing the sponsor as a barrier predicted adoption of assessments. Also, a common reason for not having adopted assessments in the past year was that sponsors already required the use of an assessment. This suggests that participants perceive sponsors as playing an important and decisive role in consent procedures. Additionally, IRBs were sometimes seen as barriers to implementing assessments by researchers, yet in qualitative interviews IRB members expressed the fewest concerns and were overall highly supportive of assessments. IRBs play an important gate-keeper role in the consent process by having the authority to approve or request modifications to consent processes to make them more ethical. If IRBs are perceived as opposing the use of assessment, researchers are less likely to implement them. IRBs may need to champion the use of assessments in order to change researcher perceptions and increase adoption going forward [49]. This is especially important because regulatory standards and guidelines are lacking [28].

### Dissemination and Implementation Efforts Will Need to Promote Very Practical Assessments

Qualitative interview participants reported that it would be burdensome to implement an assessment. They reported that adding an assessment would take additional time during an often already long and sometimes overwhelming consent process. Any intervention aimed at increasing the use of assessments may need to focus on very practical and brief assessments, and inform researchers of their brevity. For example, the UBACC can be administered in as few as 5 minutes by Bachelor’s level research staff with minimal training [8].

### Qualitative Methods Are an Important Supplement to a Survey when Studying Barriers

Notably, there were a few discrepancies between our quantitative survey data and qualitative interview data. For example, qualitative interview participants reported that time burden was a barrier to using an assessment, but survey participants did not frequently report not having time to make changes to study protocols. Additionally, qualitative interview participants reported that study participants would be a barrier to implementing assessments, but quantitative survey participants did not frequently report study participants as a barrier. These discrepancies show the need to use both qualitative and quantitative methods when investigating the barriers to implementation. Using both methods provided a fuller picture of the barriers associated with adopting validated assessments.

### Further Research Is Needed to Understand Researcher and Participant Experiences with Assessments

There are numerous benefits of routinely assessing participant understanding of consent information. However, there may be times when assessment is inappropriate. For example, researchers in the qualitative interview sample were concerned that using assessments will cause discomfort or frustration among study participants. Does an assessment do more harm than good when a potential participant appears to clearly lack decisional capacity—will it cause foreseeable embarrassment or discomfort? This is an important concern, given that the population that may be most at risk of frustration are those with undiagnosed cognitive impairments who may be unable to answer the questions and thus score poorly on the assessment. Screening for this group is one of the main reasons for using an assessment, but further research is needed to provide evidence-based methods of administering assessments in a manner that would not cause frustration.

Furthermore, it is likely that not all greater-than-minimal-risk studies require the use of an assessment. Which study designs, while technically greater than minimal risk, are so simple and safe that assessment would be an unnecessary burden? Further research is needed to identify these types of studies so that assessments can be implemented when needed, and not when they are unnecessary.

### Implementation Science Frameworks Are Crucial to Identifying and Overcoming Barriers to Adopting New Practices

Our study was guided by the CFIR implementation science framework. The majority of the barriers identified fall under the CFIR individual domain, suggesting that characteristics about researchers’ knowledge and attitudes are driving the lack of adoption of assessments. Given that attitudes and confidence were significant predictors of adopting assessments and researchers reported a lack of awareness of assessments, addressing researchers’ attitudes and knowledge is essential to increasing adoption. Thus, as already described, providing education and training on assessments will be a necessary component of any intervention aimed at increasing their use. Other barriers, such as the burden associated with implementing assessments, and seeing the research team and study participants as barriers fall under the CFIR inner domain. These are aspects internal to trials that affect the implementation of assessments. Overcoming these inner domain barriers may require training on available assessments (including those that might be low burden), education to help researchers select an assessment that would be the best fit for their team, and training on how to administer an assessment in a way that minimizes potential distress for study participants.

### Limitations

Our survey relied upon self-report data because it would have been impossible to access actual institutional records (protocols, materials, and IRB records). We controlled for socially desirable responding in some of our analyses, but it is possible that participants overreported their use of validated consent assessments. Finally, our sample was largely White and female. There are no available demographic data on CRCs in the USA; however, the sample is consistent with prior studies of CRCs and may be representative of the clinical research professional workforce in the USA [50, 51].

### Conclusion

Assessing participants understanding and appreciation of consent information are currently infrequent in the USA. Increasing the use of this consent practice is important, because both participants and research professionals frequently overestimate how well participants understand research, and experts often vary in their determinations of participants’ capacity to consent [5–8]. Furthermore, trials that are open to older adults (age 65+) or those with dementia or other cognitive impairment are particularly at risk of enrolling participants who do not understand and appreciate consent information [9–17]. Increasing the frequency with which researchers use validated assessments of consent is an important undertaking: it will increase the ethicality of clinical trials being conducted in the USA (especially as increasing number of older adults are included in the clinical research [20]) and may expedite IRB approval [27].
